# Concomitant hook of hamate fractures in patients with scaphoid fracture: more common than you might think

**DOI:** 10.1007/s00256-017-2814-3

**Published:** 2017-11-16

**Authors:** Ramin Mandegaran, Sam Gidwani, Ali Zavareh

**Affiliations:** 1 Department of Radiology, Guy’s and St Thomas’ NHS Foundation Trust, Guy’s Hospital, Great Maze Pond, London, SE1 9RT UK; 2 Department of Orthopaedics, Guy’s and St Thomas’ NHS Foundation Trust, Guy’s Hospital, Great Maze Pond, London, SE1 9RT UK

**Keywords:** Scaphoid, Hamate, Hook of hamate, Fracture

## Abstract

**Objective:**

The scaphoid is the most commonly fractured carpal bone. The presence of a concomitant hook of hamate fracture is of particular relevance given that it is often occult on routine wrist/scaphoid radiographs and that hook of hamate fractures are prone to symptomatic non-union, resulting in chronic ulnar wrist pain. Prompt diagnosis and immobilisation/fixation may minimise such complications. Our study is aimed at assessing the frequency of concomitant hook of hamate fractures in patients with scaphoid fractures.

**Methods:**

Hook of hamate fracture is often occult on wrist/scaphoid radiographs. Hence, we identified all 2,568 CT and MRI studies performed to investigate scaphoid fracture at our institution from April 2005 to March 2016. Three hundred and twelve out of 2,568 cases were confirmed to have a scaphoid fracture. Images were then retrospectively reviewed by a Consultant Musculoskeletal Radiologist and Musculoskeletal Radiologist Trainee to assess for the presence of concomitant hook of hamate fracture and, if present, whether this was identified on initial reporting.

**Results:**

Concomitant hook of hamate fracture was identified in 10.3% of cases (32 out of 312, 30 on CT, 2 on MRI); most were minimally/non-displaced. Sixty percent of fractures identified on CT were missed on the initial review (18 out of 30). Both cases identified on MRI had been initially reported.

**Conclusion:**

Scaphoid fracture is associated with higher than expected rates of concomitant hook of hamate fracture. Given the potential morbidity associated with hook of hamate fracture, this should be considered a review area when investigating scaphoid injury. These fractures are often minimally displaced, hence easily overlooked on CT. MRI may therefore be superior when investigating radiographically occult scaphoid fractures.

## Introduction

The scaphoid is the most commonly fractured carpal bone, accounting for approximately 60% of all carpal bone fractures [1, 2]. Most occur in the context of a force causing extreme dorsiflexion of the wrist—one of the classical “fall onto an outstretched hand” (FOOSH) injuries.

Previous studies assessing rates of concomitant fractures associated with scaphoid injury have been limited in number and scope, but have described a concomitant fracture rate of between 5 and 13%, typically involving the distal radius, and to a lesser extent, the triquetrum, capitate, hamate (non-hook), metacarpals and phalanges [1, 3, 4]. However, these studies were limited to plain radiographic assessment of scaphoid injury. In our institution, CT and MRI studies are often performed in the context of scaphoid injury (e.g., assessment of radiographically occult injury, surgical planning, assessment of complications and healing), and several concomitant hook of hamate fractures were noted on routine reporting of cross-sectional studies positive for scaphoid fractures—an association not described in previous epidemiological studies. A literature review has identified only one case report of a hook of hamate fracture associated with a scaphoid waist fracture [5]. The purpose of this study was to formally assess the rate of concomitant hook of hamate fractures in a large cohort of patients with cross-sectional studies positive for scaphoid fracture, and to determine the presence of an association between scaphoid injury and hook of hamate fractures.

## Materials and methods

In a retrospective analysis, all CT and MRI studies performed to investigate scaphoid injury at a single tertiary centre between April 2005 and March 2016 were analysed for the presence of a scaphoid fracture. Of a total of 2,568 cases, 312 patients were identified to have CT- or MRI-proven scaphoid fracture (265 CT scans and 47 MRI scans). Images were then retrospectively reviewed by both a consultant musculoskeletal radiologist and a musculoskeletal radiologist trainee to identify the presence of a concomitant fracture of the hook of hamate. For each concomitant hook of hamate fracture identified, the degree of fracture displacement was recorded in line with accepted definitions typically applied to scaphoid fracture, such that fractures were categorised as either non-displaced/minimally displaced (up to 1 mm displacement) or displaced (greater than 1 mm displacement) [6, 7]. We further subcategorised displaced fractures as those between 1- and 2-mm displacement, and those with greater than 2-mm displacement. Whether the fracture involved the tip, waist or base of the hook of hamate was also recorded. Plain radiographs of all positive cases were also subsequently reviewed to determine whether concomitant hook of hamate fractures were radiographically discernible retrospectively.

## Results

Of all 312 patients with a scaphoid fracture confirmed on cross-sectional imaging, 32 (10.3%) were identified with concomitant hook of hamate fractures on CT or MRI. Of these 32 patients, 30 were identified from the cohort of 265 CT studies; the remaining 2 were identified from the 47 MRI scans. Of the 30 cases of concomitant hook of hamate identified on CT, only 12 (40%) were identified on initial reporting (Table 1), although none of the three fractures displaced by more than 2 mm were missed. Of the remaining 18 CT studies positive for hook of hamate fracture, which were not identified on initial reporting, most were non-displaced/minimally displaced (15 patients; Table 2). Both concomitant hook of hamate fractures identified on MRI were also identified on initial reporting. Of all 32 concomitant hook of hamate fractures identified, only 4 were displaced >2 mm.

**Table 1:** Total numbers of concomitant hook of hamate fractures identified on CT and MRI during the study and on initial reports

	Total number of patients with scaphoid fracture	Total number of concomitant hook of hamate fracture identified during the study	Total number of concomitant hook of hamate fractures initially reported
CT	265	30	12
MRI	47	2	2
Total	312	32	14

**Table 2:** Summary statistics of concomitant hook of hamate fractures identified according to modality and whether reported or missed on initial reporting. These have been subcategorised according to the degree of displacement and location of the hook of hamate fracture

		Displacement			Total	Location of fracture			Total
		Non-displaced/minimally displaced (≤1 mm)	Displaced 1-2 mm	Displaced >2 mm		Tip	Waist	Base	
CT	Reported	5	4	3	12	1	4	7	12
	Missed	16	2	0	18	6	3	9	18
MRI	Reported	0	1	1	2	0	0	2	2
	Missed	0	0	0	0	0	0	0	0
Total		21	7	4	32	7	7	18	32

Most of the concomitant hook of hamate fractures involved the base of the hook (18 out of 32), whereas the remainder involved either the tip or waist in equal proportions (7 out of 32 respectively). None of the hook of hamate fractures were comminuted. The proportion of hook of hamate fractures that were originally reported was greatest for waist fractures (57%), followed by fractures of the base (39%) and tip (14%; Table 2). None of the hook of hamate fractures was confidently identifiable on retrospective review of plain radiographs performed before cross-sectional imaging.

## Discussion

Detection of concomitant injuries in addition to scaphoid fracture is essential to ensure that the complete injury profile is taken into consideration when planning subsequent management. Concomitant hook of hamate fractures are of particular relevance given that they are often minimally displaced and both clinically and radiographically difficult to detect; yet, they are prone to poor healing and non-union owing to the combination of a tenuous blood supply, and persistent traction from tendinous and ligamentous attachments (opponens digiti minimi and flexor digiti minimi brevis tendons, pisohamate ligament, distal end of the transverse carpal ligament) [8–10]. Chronic symptomatic non-union (including chronic ulnar-sided wrist pain, reduced grip strength, ulnar neuropathy secondary to nerve compression in Guyon’s canal) frequently requires excision of the non-united hook fragment [8, 11].

Investigation of scaphoid injury has traditionally been limited to a dedicated four-view plain radiographic “scaphoid series”, often comprising: posterior–anterior (PA) view with ulnar deviation; lateral view; semi-supinated oblique; and semi-pronated oblique views [12, 13]. However, given the potential complications associated with missed diagnoses (non-union, mal-union and avascular necrosis) and the increasing availability of prompt cross-sectional imaging, MRI and CT are increasingly being utilised to investigate cases of suspected radiographically occult scaphoid injury, provide an aid to surgical planning and assess for the presence of healing or complications. Our study demonstrates a higher than expected rate of concomitant hook of hamate fractures identified on cross-sectional studies of patients with scaphoid fracture—an association that has not been recognised in any previous epidemiological studies assessing scaphoid injuries [1–4, 14–16]. This is perhaps not surprising given that these previous studies have focused on plain radiographic assessment of scaphoid injury, a modality on which detection of hook of hamate fractures is notoriously difficult [17–19]. Indeed, none of the 32 hook of hamate fractures we identified was discernible on retrospective review of corresponding plain radiographs, a finding supported by a previous study of 10 patients with subsequently confirmed hook of hamate fracture, none of which was detectable on standard PA, lateral and oblique wrist radiographic projections (dedicated carpal tunnel views demonstrated the fractures in 8 patients) [17].

This frequency of association of hook of hamate fractures with scaphoid fractures is also perhaps a little surprising, as hamate hook fractures are generally thought of as injuries usually sustained through sports such as golf or tennis, by the impact of a racket or club against the hamate. However, the hook of hamate can also be injured by indirect means via a fall on the outstretched hand [20], and the subset of fractures seen in this series are more likely due to this mechanism via avulsion of the hook by its ligamentous attachments, as the wrist is forced into dorsiflexion.

In the context of known scaphoid fracture, knowledge of the presence of a concomitant hook of hamate fracture would have implications for the patient’s management. First, in the context of percutaneous scaphoid fracture fixation or open reduction and internal fixation, the patient can be mobilised 2 weeks after fixation. However, knowledge of a concomitant hook of hamate fracture would prolong the length of time the patient is immobilised, given that hook of hamate fractures are typically managed conservatively with immobilisation for 6 weeks [21]. Second, if treating a scaphoid fracture conservatively with a cast, knowledge of a concomitant hook of hamate fracture would alert the clinician to examine the hamate following cast removal, and repeating cross-sectional imaging at 3 months to assess healing. Third, the patient with a concomitant hook of hamate fracture could also be counselled regarding the risks of non-union, particularly if the fracture is displaced. Although the risk of hook non-union is difficult to quantify (given that most are identified as delayed or non-unions rather than acute injuries), one recent retrospective study described a non-union rate of 24% amongst their cohort of 25 patients, with hook of hamate fractures treated conservatively with immobilisation [22].

**Fig. 1:**
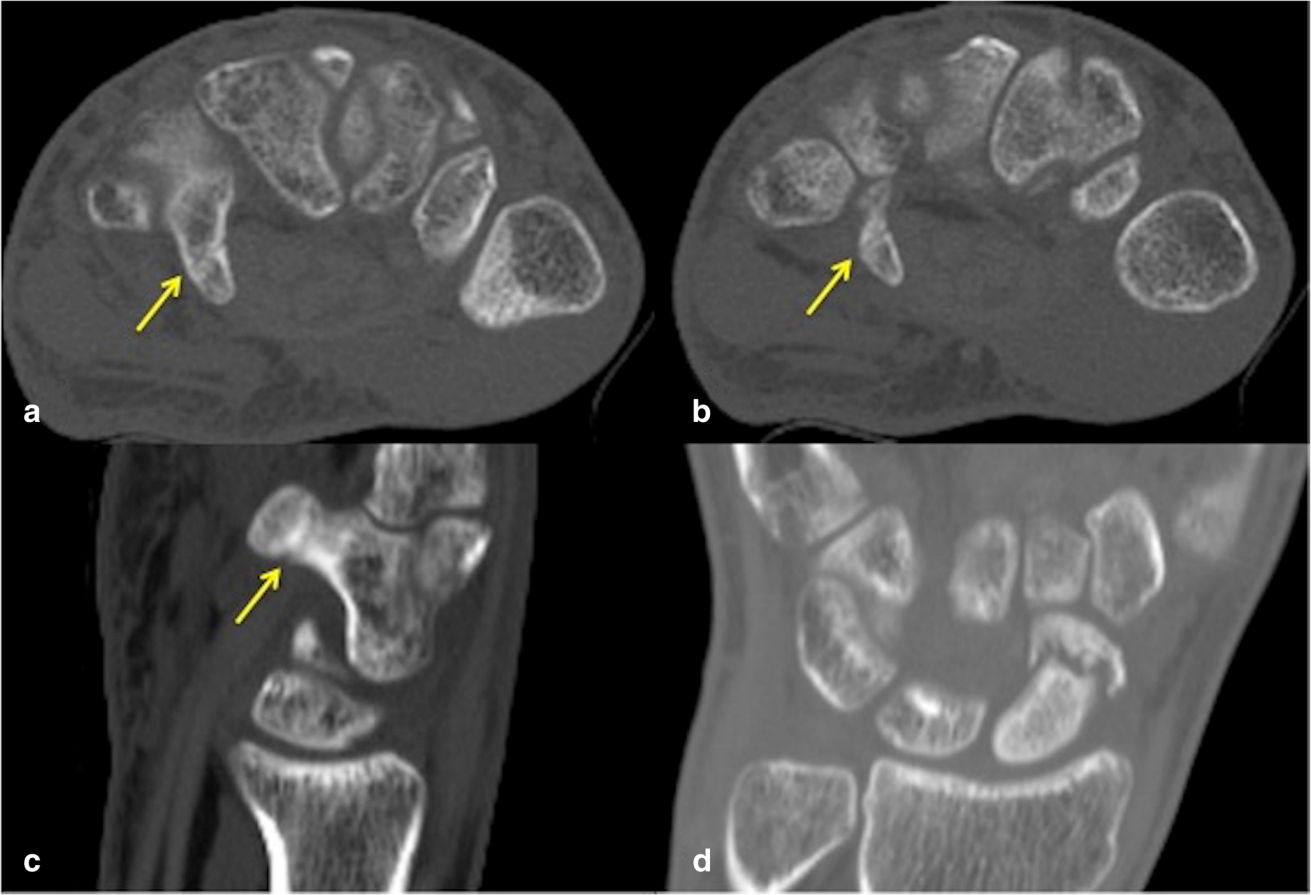
A 24-year-old man with fracture of the right scaphoid and concomitant hook of hamate fracture (CT performed 3 months after initial injury). **a** Axial CT bone window shows the subtle non-displaced fracture line across the hook of hamate waist (*arrow*), also seen in **b** at a slightly more distal level. Minimally and non-displaced hook of hamate fractures are often most conspicuous on axial images followed by **c** sagittal reformats. Note the marginal sclerosis in **a–c** suggestive of fracture healing. Interval CT 4 months later (not shown) confirmed a healed hook of hamate fracture. **d** Coronal reconstruction demonstrates the poorly healing distal pole scaphoid fracture with some sclerosis of the proximal component

**Fig. 2:**
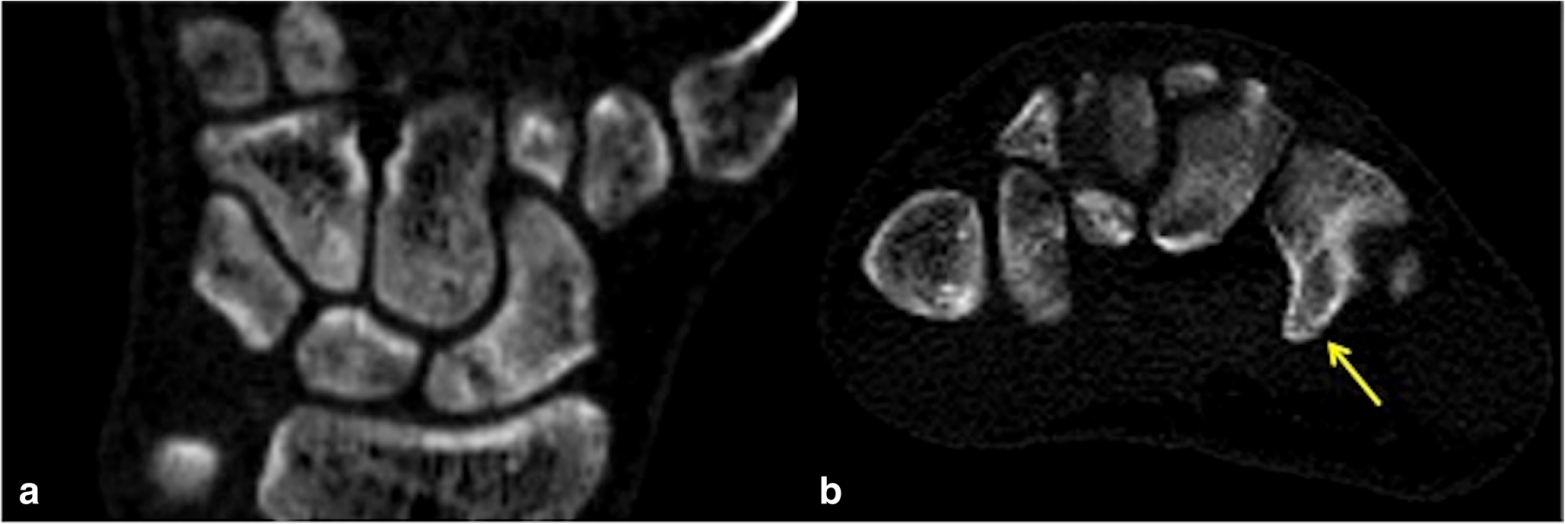
A 24-year-old man with fracture of the left scaphoid and concomitant hook of hamate fracture involving the tip of the hamate hook (CT performed 2 weeks after the initial injury). **a** Coronal CT reconstruction shows the minimally displaced fracture of the proximal pole of the scaphoid with some resorption of the fracture margins. **b** Axial CT image shows the subtle non-displaced concomitant fracture of the left hook of hamate tip (*arrow*), which is easily overlooked if not actively sought

Given that 60% of hook of hamate fractures in our study were not identified at the time of initial CT reporting, this highlights the difficulty in detecting hook of hamate fractures in the routine reporting of CT studies in the absence of the observer being made aware of any clinical suspicion of hamate injury. Indeed, most hook of hamate fractures identified in our study were non-displaced and subtle findings, which on CT, were identified only following dedicated review of the hamate on axial, coronal and sagittal multiplanar reformats as part of the study protocol (Figs. 1, 2). We found the axial reformats to be the most useful, followed by review of the sagittal images. The coronal reformats were of little help given the en-face orientation of most hook of hamate fractures in the coronal plane.

**Fig. 3:**
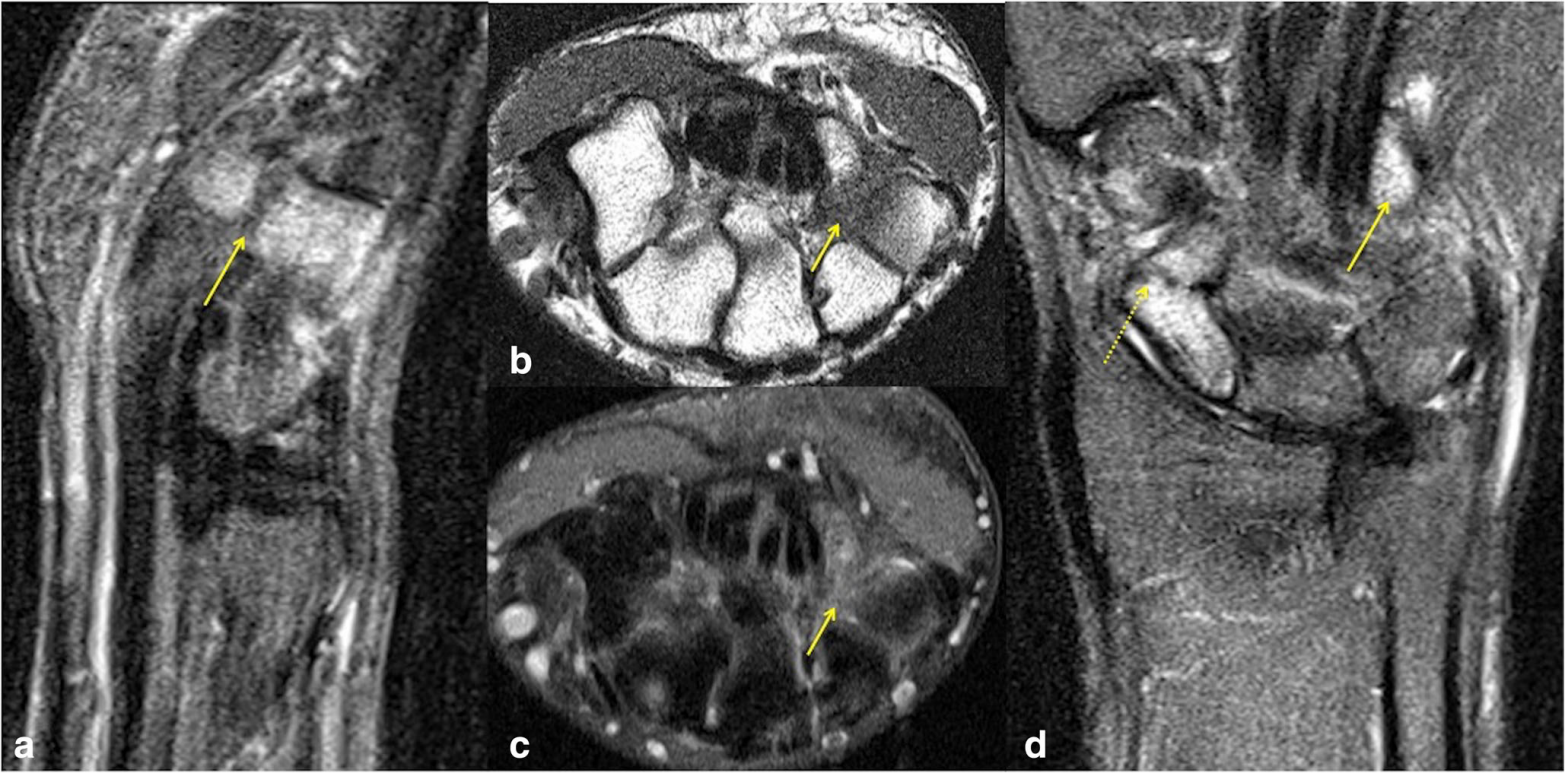
A 21-year-old man with fracture of the left scaphoid and concomitant hook of hamate fracture. **a** Sagittal STIR shows significant marrow oedema involving the hook and body of the hamate, which is easily discernible compared with the low signal observed in the other imaged bones. A low signal fracture line is seen across the base of the hook of hamate (*arrow*). **b** Hook of hamate fracture on axial T1 and **c** corresponding axial STIR sequences, both demonstrating obvious marrow signal abnormality, although the displaced fracture line is more conspicuous on T1. **d** Coronal STIR sequences easily demonstrate marked oedema with angulated fracture of the distal scaphoid pole (*dashed arrow*), but also draw attention to marked marrow oedema of the hook of hamate (*arrow*). As is generally the rule with MRI of most fractures, bony abnormality was most notable on the fluid-sensitive sequences due to marrow oedema, although the fracture line was most conspicuous on T1

Considerably fewer patients were investigated with MRI compared with CT, in part because of the easier accessibility of prompt CT in our institution. However, in the only two MRI studies, where concomitant hook of hamate fractures were identified, both were also identified on initial reporting. This is perhaps of little surprise, given that on MRI, marrow signal alteration surrounding a recent fracture is easily observed, regardless of the extent of the fracture. The fracture usually manifests as a well-defined low-signal line on a background of surrounding marrow oedema-related signal abnormality (Fig. 3), and is usually markedly more conspicuous than any corresponding CT findings [23]. This highlights the point that MRI is the most reliable imaging modality for identifying minimally/non-displaced fractures such as the scaphoid or hook of hamate, and that it is the modality of choice for detection of any radiographically occult concomitant fractures in patients with scaphoid fracture [23, 24].

However, owing to constraints of availability and the cost of MRI compared with CT in many centres, CT is frequently performed in the context of suspected/proven scaphoid injury. This is particularly true in two contexts: that of surgical planning, where CT depiction of bony anatomy is often preferred to MRI as a visual aid by orthopaedic surgeons; and in the assessment of scaphoid healing, where CT is the modality of choice. Previous CT studies have fortunately demonstrated the high level of sensitivity of CT for hook of hamate fractures, where the diagnosis is considered [25].

Of the 18 patients in whom the hook of hamate fracture was not identified at initial CT reporting, 1 patient continued to have persistent symptoms at follow-up in the context of a healed scaphoid fracture but persistent hook of hamate fracture at interval CT. Specifically, interval CT 3 months post-injury showed a healed scaphoid tubercle fracture but persistent non-displaced hook of hamate fracture (again not identified on initial reporting). Clinically, follow-up information was available up to 5 months post-injury, describing persistent volar wrist pain (albeit markedly improved since the initial injury) and reduced grip strength. Although the on-going symptoms in this case cannot with any certainty be attributed to a persistent non-united hook of hamate fracture, in the absence of any other significant CT finding, it is a likely explanation.

## Conclusion

Scaphoid fractures are associated with a higher than expected rate of concomitant fractures of the hook of hamate (10.3% in our study). These concomitant hook fractures are often either non-displaced or only minimally displaced, and were all occult on conventional radiography. Hook of hamate fractures are associated with a risk of poor healing and non-union, leading to chronic palmar/ulnar-sided wrist pain. In the context of coexistent scaphoid fracture, alerting the clinician to the presence of a concomitant hook of hamate fracture may have implications for optimal management. MRI is the most suitable modality for identifying minimally or non-displaced hook of hamate fractures, whereas on CT the findings are often subtle and easily overlooked. We recommend that the hook of hamate be considered a review area when reporting any cross-sectional studies demonstrating scaphoid fracture.
